# BRCA Mutations and MicroRNA Expression Patterns in the Peripheral Blood of Breast Cancer Patients

**DOI:** 10.1021/acsomega.3c10086

**Published:** 2024-04-03

**Authors:** Ceren Alavanda, Esra Dirimtekin, Maria Mortoglou, Esra Arslan Ates, Ahmet Ilter Guney, Pinar Uysal-Onganer

**Affiliations:** † Department of Medical Genetics, School of Medicine, Marmara University, 34854 Istanbul, Turkey; ‡ Department of Medical Genetics, Van Research and Training Hospital, 10300 Van, Turkey; § Cancer Mechanisms and Biomarkers Research Group, School of Life Sciences, University of Westminster, W1W 6UW London, U.K.; ∥ Department of Medical Genetics, Istanbul University-Cerrahpasa, Cerrahpasa Faculty of Medicine, 34098 Istanbul, Turkey

## Abstract

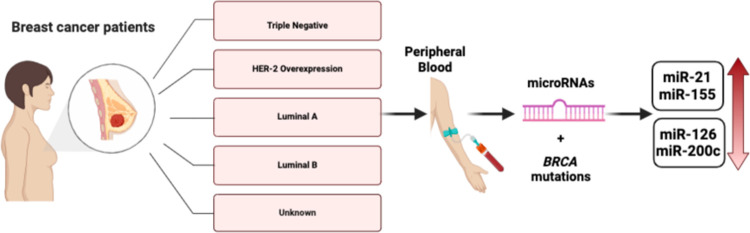

Breast cancer (BC) persists as the predominant malignancy globally, standing as the foremost cause of cancer-related mortality among women. Despite notable advancements in prevention and treatment, encompassing the incorporation of targeted immunotherapies, a continued imperative exists for the development of innovative methodologies. These methodologies would facilitate the identification of women at heightened risk, enhance the optimization of therapeutic approaches, and enable the vigilant monitoring of emergent treatment resistance. Circulating microRNAs (miRNAs), found either freely circulating in the bloodstream or encapsulated within extracellular vesicles, have exhibited substantial promise for diverse clinical applications. These applications range from diagnostic and prognostic assessments to predictive purposes. This study aimed to explore the potential associations between *BRCA* mutations and specific miRNAs (miR-21, miR-155, miR-126, and miR-200c) expression that are known to be dysregulated in BC patient samples. Our findings indicate a robust correlation between miRNA expression status and disease subtypes. We found a correlation between the expression status of miRNAs and distinct disease subtypes. Intriguingly, however, no significant associations were discerned between disease status, subtypes, or miRNA expression levels and the presence of *BRCA* mutations. To advance the validation of miRNAs as clinically relevant biomarkers, additional investigations within larger and meticulously selected patient cohorts are deemed imperative. These microRNA entities hold the potential to emerge as groundbreaking and readily accessible tools, poised for seamless integration into the landscape of clinical practice.

## Introduction

Breast cancer (BC) remains the most prevalent cancer worldwide and is a leading cause of cancer-related mortality among women.^1^ Currently, the classification and assessment of BC primarily rely on tumor staging, grading, and several molecular biomarkers, including estrogen receptor (ER), progesterone receptor (PgR), human epidermal growth factor receptor 2 (HER2), and *K*_i_-67 (a proliferation marker). Based on the expression of these markers, BC is categorized into four primary subtypes, each with distinct prognoses and outcomes: luminal A (ER+, PR+, HER2–, low *K*_i_-67), luminal B (ER+, PR±, HER2±, high *K*_i_-67), HER2 overexpression (ER–, PR–, HER2+), and triple-negative (TNBC/ER–, PR–, HER2−).^2^ Around 40–50% of BCs are of the Luminal A subtype, while Luminal B, HER2 overexpression, and triple-negative frequencies are approximately 20–30, 15–20, and 10–20%, respectively.^3^ In the Luminal A subtype of BC; patients generally experience a favorable prognosis, with higher chances of overall survival. In contrast, the prognosis for the Luminal B subtype is moderate; however, in TNBC and HER2 overexpression subtypes, the prognosis is notably poorer, suggesting a higher level of challenge and a lower probability of overall survival.^3^ Similar to other malignancies, the etiology of BC is multifactorial and complicated. Although most cases are sporadic, 5–10% are hereditary.^4^ The most common cause of hereditary BC is germ line pathogenic variants (PVs) in the *BRCA1* and *BRCA2* genes. PVs in these genes show a highly penetrant autosomal dominant inheritance.^5^ Approximately 50 to 65% of women with a PV in *BRCA1*, and 40 to 57% of women with a PV in *BRCA2* will develop BC by age 70, respectively.^5^*BRCA1* and *BRCA2* genes are involved in homologous recombination repair; therefore, they act as tumor suppressor genes. The *BRCA1* gene is located at 17q21 and has 22 exons. The *BRCA2* gene is located at the 13q13 and has 27 exons. So far, over 3000 pathogenic variants have been discovered in either the *BRCA1* or *BRCA2* genes. Individuals carrying *BRCA1* PVs are at a higher risk of developing TNBC compared to those without *BRCA1* PVs.^6^ Additionally, individuals with *BRCA1* PVs tend to have lower levels of ER, higher histological grades, and a greater proliferation index. In contrast, individuals carrying *BRCA2* PVs are more likely to have ER+ BC.^7^ Whether a *BRCA* mutation in BC is linked to an unfavorable prognosis is still a subject of debate and disagreement among experts. Studies have consistently shown an elevated risk of contralateral BC in patients with *BRCA* PVs. On the other hand, whether the risk of ipsilateral BC is higher in women with *BRCA* PVs remains controversial.^7^

MicroRNAs (miRNAs/miRs) are small noncoding RNA molecules known to play a crucial role in regulating gene expression in eukaryotic cells. Typically consisting of 21 to 25 nucleotides, miRNAs regulate post-transcriptional gene expression by binding to mRNA. This binding primarily occurs at the target mRNA’s 3′ untranslated region (3′UTR). Here, miRNAs can either inhibit protein translation or initiate the degradation of target mRNAs. By modulating the expression of specific genes, miRNAs play a crucial role across a broad spectrum of biological processes, such as cellular differentiation apoptosis and responses to environmental changes and stressors. Abnormal miRNA expressions or functions have been associated with various diseases, including cancer, neurodegenerative, cardiovascular, and metabolic disorders.^8,9^

In this study, we have selected miRNAs with established clinical verification from the literature, aiming to explore their potential correlation with *BRCA* mutations.^10,11^ Among these miRNAs, miR-155 and miR-21 fall into the category of oncomiRs, while miR-126 and miR-200c have been demonstrated to function as tumor suppressors. miR-21, one of the most studied oncologic miRNAs, has been reported to be highly expressed in several malignancies compared to corresponding normal tissues.^12−14^ Furthermore, elevated levels of circulating cell-free miR-21 have been associated with poor prognostic outcomes in specific cancers such as breast, prostate, colon, and pancreas.^8,15−20^ In BC, it has been demonstrated that reported adverse patient prognoses are linked to either increased expression levels of circulating miR-21 or increased tumor expression of miR-21.^21−25^ Moreover, upregulation of miR-21 in neoplastic cells of hormone receptor-positive cancers correlated with poor prognosis, whereas elevated stromal levels of miR-21 were associated with worse outcomes for patients with triple-negative BC.^26,27^ miR-155 is another example of oncogenic miRNAs, which is associated with clinicopathologic markers, tumor subtype, and poor survival rates in BC. Additionally, miR-155 overexpression is linked to both invasiveness and recurrence of breast tumors, while miR-155 target genes are of potential clinical prognostic value.^28^ Specifically, miR-155 upregulation is associated with high tumor grade, advanced stage, and lymph node metastasis.^29^ In BC, lower expression levels of miR-200c have been associated with poor overall survival and disease-free survival.^30^ Particularly, miR-200c downregulation has been found in both TNBC tissues and BC cells, and therefore, it could be used as a valuable marker for BC progression and prognosis.^31^ It has been reported that miR-126 expression levels are lower in ER+ BC and ductal carcinoma *in situ* breast tissues as compared to normal adjacent ones, and this downregulation is correlated with shorter overall survival.^32−34^ Loss of miR-126 expression in BC tissue has also been related to poor distal metastasis-free survival, while restoration of miR-126 suppresses overall tumor growth and proliferation.^35^

Despite significant advancements in prevention and treatment, including targeted and immunotherapies, there remains a need for new tools to identify women at higher risk of BC. Liquid biopsies became an important tool for biomarker testing, and therefore, in this study, we aimed to test the potential of using selected miRNAs as biomarkers in BC patient samples and to elucidate their possible associations with *BRCA* mutations.

## Results

We examined the expression levels of miR-21, miR-155, miR-126, and miR-200c in BC patients exhibiting various clinical characteristics. Additionally, we conducted an analysis of the *BRCA* status within the same cohort to explore potential connections between the *BRCA* status and the expression of these selected miRNAs in 48 peripheral blood BC samples.

**Figure 1:**
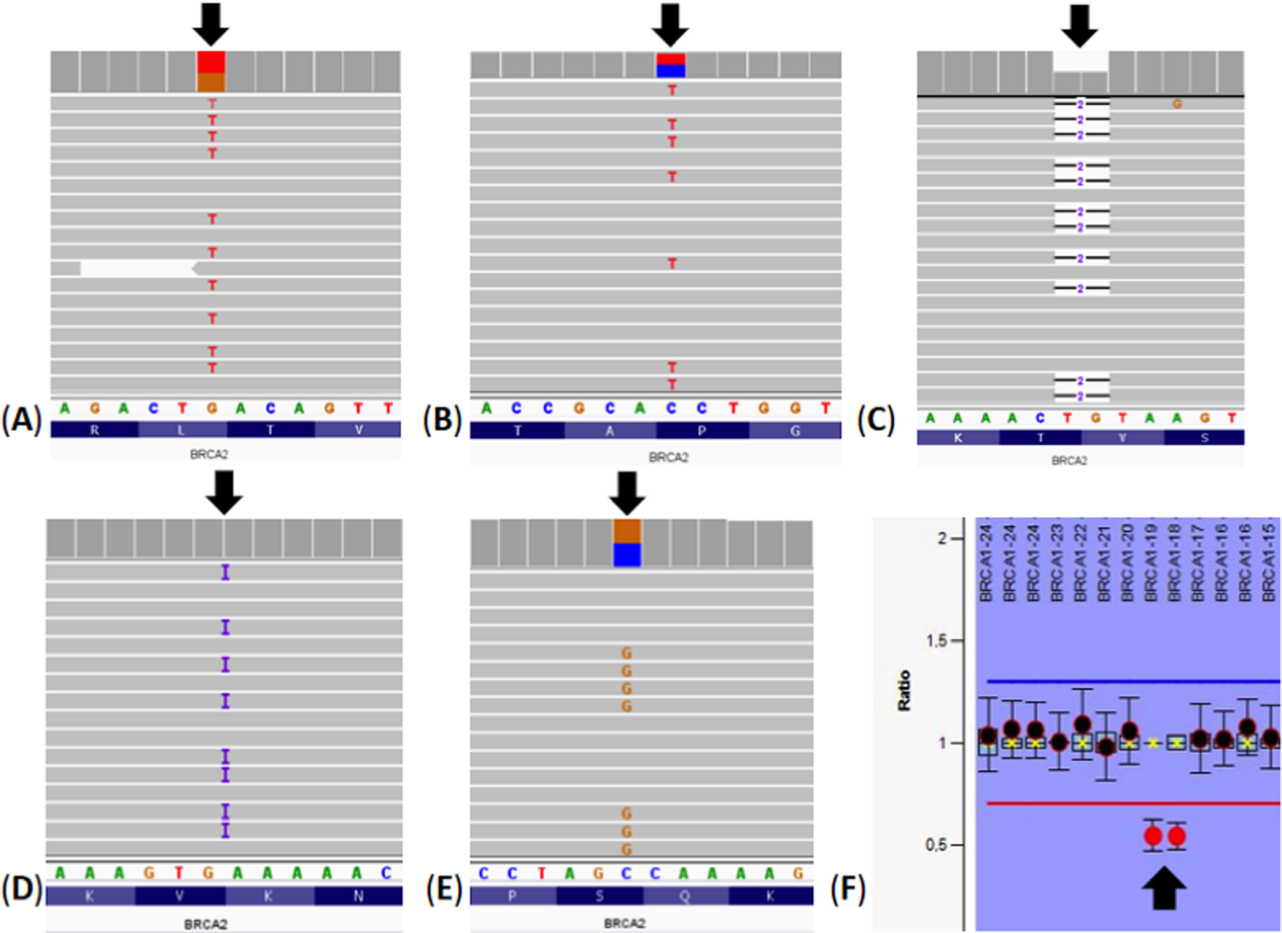
Integrative Genomics Viewer (IGV) visualization of the detected variants and MLPA data of copy number variants in *BRCA1*/*2* genes. (A) A heterozygous c.8235G > T (p.Leu2745Leu) variant in the *BRCA2* gene. (B) A heterozygous c.7054C > T (p.Pro2352Ser) variant in the *BRCA2* gene. (C) A heterozygous c.3847_3848delGT (p.Val1283Lysfs*2) variant in the *BRCA2* gene. (D) A heterozygous c.4631dupA (p.Asn1544Lysfs*4) variant in the *BRCA2* gene. (E) A heterozygous c.3318C > G (p.Leu2745Leu) variant in the *BRCA2* gene. (F) A heterozygous exon 18–19 deletion in the *BRCA1* gene.

**Figure 2:**
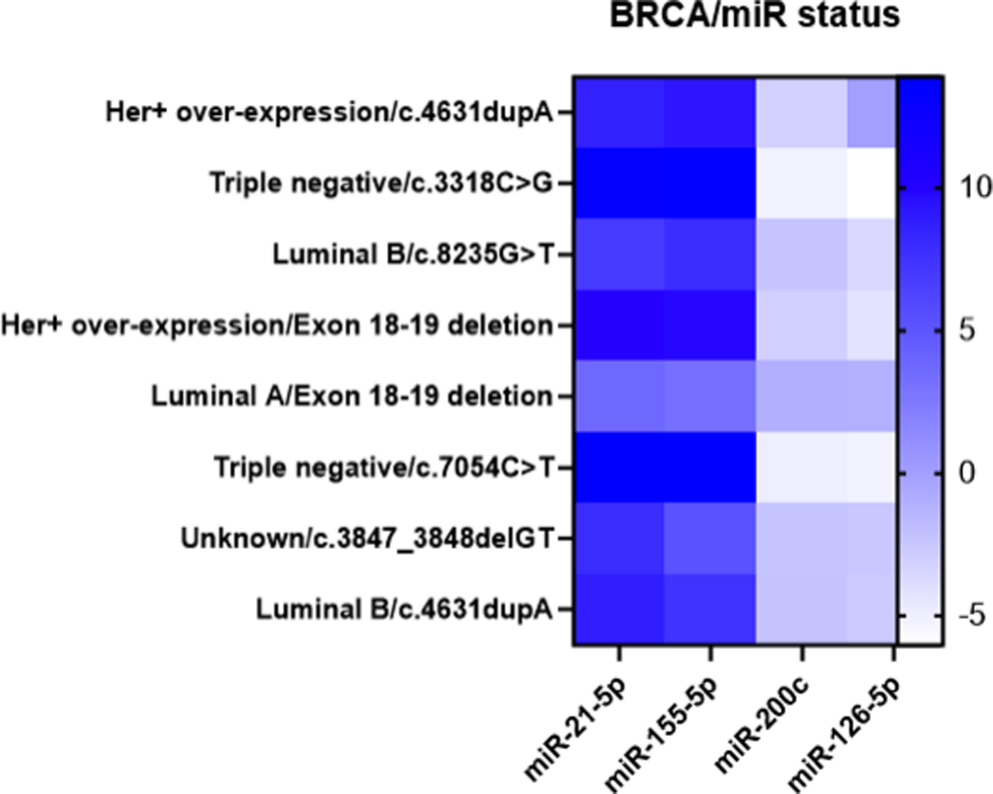
*BRCA* mutations and miRNA expression patterns in BC blood samples. Heatmap shows the expression levels of miR-21, miR-155, miR-126, and miR-200c correlated to *BRCA* mutations in different BC subtypes (HER+overexpression, TNBC, Luminal A/B, and unknown).

In 7 out of 48 patients (14.5%), PVs and variants of uncertain significance (VUS) were detected in the *BRCA* genes. In one patient, a variant was detected in the *BRCA1* gene; in five patients, variants were found in the *BRCA2* gene; and in one patient, variants were identified in both the *BRCA1* and *BRCA2* genes. The patient with variants in both *BRCA1* and *BRCA2* had a pathogenic variant in *BRCA1*, while the variant in *BRCA2* was a novel VUS variant. Two unrelated patients carried the same pathogenic *BRCA1* variant (heterozygous exon 18–19 deletion). Another two unrelated patients carried the same pathogenic known variant (c.4631dupA, p.Asn1544Lysfs*4) in the *BRCA2* gene. Among the detected variants in the *BRCA2* gene, two were known pathogenic variants, one was known VUS, and two were novel VUS (c.7054C > T, p.Pro2352Ser; c.8235G > T, p.Leu2745Leu). All pathogenic variants in the *BRCA2* gene are truncating variants. However, all VUS variants in the *BRCA2* gene were missense variants. Integrative Genomics Viewer (IGV) visualization of the detected variants and MLPA data of copy number variants in *BRCA1/2* genes are shown in Figure 1. Figure 2 represents the *BRCA* mutations (c.4631dupA, c.3318C > G, c.8235G > T, Exon 18–19 deletion, c.7054C > T and c.3847_3848delGT) in the peripheral blood samples of BC cases and microRNA expression patterns.

Of the five patients carrying variants in the *BRCA2* gene, two had luminal B (40%), two had HER2 overexpression (40%), and one had TN (20%) BC. The patient with a PV in the *BRCA1* gene had triple-negative BC, whereas the patient with variants detected in both *BRCA1* and *BRCA2* had HER2 overexpression BC. None of the patients with Luminal A subtype had any variants detected in the *BRCA* genes. Among the 19 patients with lymph node involvement, one had a VUS in the *BRCA2* gene and one had a PV in the *BRCA1* gene. Among the seven patients with metastasis, only one had a VUS detected in the *BRCA2* gene. In two patients with bilateral BC and in the patient with both breast and ovarian cancers, no variants were detected in the *BRCA* genes. In the patient with recurrent (ipsilateral) BC, PV was detected in the *BRCA2* gene. The age of BC diagnosis, molecular subtypes, TNM staging, and *BRCA* statuses of the patients are summarized in Table 1.

**Table 1:** Clinical and Pathological Features of BC Patients Categorized by Their *BRCA* Variants Status Mapped with Age of Diagnosis, TNM Staging, and Molecular Subtypes

				BRCA status					
patient no.	diagnosis/age	molecular subtype	TNM staging	gene	Zygosity	variant	gnomAD	Clinvar	ACMG
1	UBC/38	Her+ overexpression	T2N2bM0	-	-	-	-	-	-
2	UBC/73	Triple negative	T1cN0M0	-	-	-	-	-	-
3	UBC/49	Triple negative	T1N0M0	-	-	-	-	-	-
4	UBC/44	Luminal A	T2N0M0	-	-	-	-	-	-
5	UBC/39	Her+ overexpression	T2N0M0	*BRCA2*	Het.	c.4631dupA (p.N1544Kfs^a^4)	<0.01%	P	P
6	UBC/33	Luminal B	Unknown	-	-	-	-	-	-
7	UBC/46, GC/46	Luminal B	Unknown	-	-	-	-	-	-
8	UBC/47	Triple negative	T4N2M1	-	-	-	-	-	-
9	UBC/59	Luminal B	T1cN1aM0	-	-	-	-	-	-
10	UBC/48	Unknown	T1bN0M0	-	-	-	-	-	-
11	UBC/48	Luminal A	Unknown	-	-	-	-	-	-
12		Unknown	Unknown	-	-	-	-	-	-
13	BBC/65	Luminal B	T1cN0M0	-	-	-	-	-	-
14	BBC/44–50	Luminal B	T1bN0M0	-	-	-	-	-	-
15	UBC/43	Luminal A	T2N0M0	-	-	-	-	-	-
16	UBC/43	Unknown	Unknown	-	-	-	-	-	-
17	UBC/45	Luminal A	T2N1M0	-	-	-	-	-	-
18	UBC/67	Luminal A	T1cN0M0	-	-	-	-	-	-
19	UBC/44	Triple negative	T2N1M1	*BRCA2*	Het.	c.3318C > G (p.S1106R)	<0.01%	VUS(5), LB(1)	VUS (PM2)
20	UBC/35	Luminal B	Unknown	-	-	-	-	-	-
21	UBC/45	Luminal B	T2N1M0	-	-	-	-	-	-
22	UBC/63	Luminal B	T1cN1M0	-	-	-	-	-	-
23	UBC/42	Luminal B	T1cN0M0	-	-	-	-	-	-
24	UBC/64	Unknown	T1cN1M0	-	-	-	-	-	-
25	UBC/43	Luminal A	T1cN0M1	-	-	-	-	-	-
26	UBC/39	Luminal B	T1cN0M0	-	-	-	-	-	-
27	UBC/41	Unknown	T2NXMX	-	-	-	-	-	-
28	UBC/47	Luminal B	T2N0M0	-	-	-	-	-	-
29	UBC/47	Luminal B	TXN0M0	*BRCA2*	Het.	c.8235G > T (p.L2745L)	N/A	-	VUS (PM2, BP7)
30	UBC/54	Luminal B	T1cN3M1	-	-	-	-	-	-
31	UBC/35	Luminal B	T2N2aM1	-	-	-	-	-	-
32	UBC/59, CML/56	Triple negative	TXN3M0	-	-	-	-	-	-
33	UBC/39	Her+ overexpression	T1cN2aM1	-	-	-	-	-	-
34	UBC/50	Luminal B	T1N2M0	-	-	-	-	-	-
35	UBC/50	Luminal B	T2N3M1	-	-	-	-	-	-
36	UBC/44	Luminal B	Unknown	-	-	-	-	-	-
37	UBC/44	Her+ overexpression	T2N0M0	*BRCA1*	Het.	Exon 18–19 deletion	<0.01%	P	P
				*BRCA2*	Het.	c.7054C > T (p.P2352S)	N/A	-	VUS (PM2)
38	UBC/66	Triple negative	T1N2M0	*BRCA1*	Het.	Exon 18–19 deletion	<0.01%	P	P
39	UBC/64	Luminal A	T1cN1M0	-	-	-	-	-	-
40	UBC/42	Luminal B	T2N1aM0	-	-	-	-	-	-
41	UBC/39	Unknown	Unknown	-	-	-	-	-	-
42	UBC/56, OC/60	Unknown	Unknown	-	-	-	-	-	-
43	UBC/48	Luminal B	T1cN1aM0	-	-	-	-	-	-
44	RBC/47–58	Her+ overexpression	Unknown	*BRCA2*	Het.	c.3847_3848delGT (p.V1283Kfs^a^2)	0.04%	P	P
45	UBC/55	Unknown	T1N2M0	-	-	-	-	-	-
46	UBC/33	Unknown	Unknown	-	-	-	-	-	-
47	UBC/39	Luminal B	Unknown	*BRCA2*	Het.	c.4631dupA (p.N1544Kfs^a^4)	<0.01%	P	P
48	UBC/45	Luminal B	T2N0M0	-	-	-	-	-	-

a Population frequencies have been calculated according to the gnomAD, ExAC, and ESP6500 databases. UBC: Unilateral breast cancer, BBC: Bilateral breast cancer (contralateral), RBC: Recurrent breast cancer (ipsilateral), GC: Gastric cancer, OC: Ovarian cancer, CML: Chronic myeloid leukemia, het: heterozygous, P: Pathogenic, VUS: Variant of uncertain significance, LB: Likely benign, N/A: Not available, Refseq: *BRCA1* (NM_007294.4), *BRCA2* (NM_000059.4).

**Figure 3:**
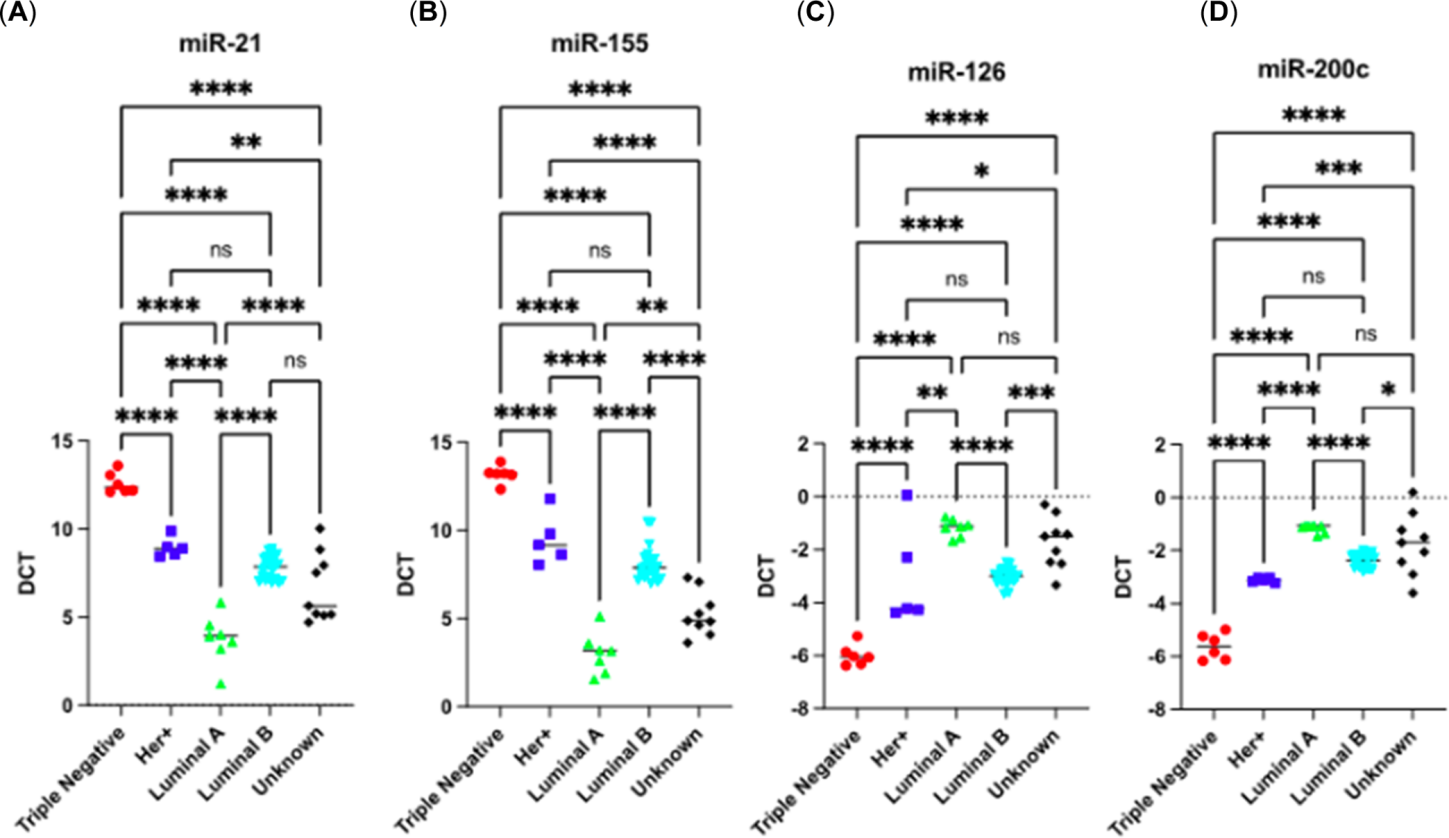
Comparative reverse transcription quantitative polymerase chain reaction (RT-qPCR) analysis of expression levels miR-21, miR-155, miR-126, and miR-200c. (A) miR-21 relative expression levels; (B) miR-155 relative expression levels; (C) miR-126 relative expression levels; (D) miR-200c relative expression levels. TNBC patients exhibit the highest levels of miR-21 and 155 and the lowest expressions of miR-126 and miR-200c. This pattern is followed by HER+ and Luminal A and B BC subtype patients. The column graphs represent the average of three replicates of RNA isolated from each sample. Data normalized according to RNU6 expression by fold analysis (*n* = 3, *p* < 0.05 for all). Exact *p*-values are indicated (* *p* ≤ 0.05; ** *p* ≤ 0.01; *** *p* ≤ 0.001; **** *p* ≤ 0.0001); error bars indicate standard deviation (SD).

We then investigated the expression levels of miR-21 and miR-155 as oncomiRs and those of miR-126 and miR-200c as tumor suppressor miRNAs. We found that TNBC patients have the highest miR-21 and 155 followed by HER+ and Luminal A and B BC subtype patients (Figure 3A,B). Tumor suppressor miRNAs miR-126 and miR-200c were found to be expressed highest on the Luminal A subtype of BC. The lowest miR-126 and miR-200c expressions were found in patients with TNBC followed by HER+ BC subtypes (Figure 3C,D). Interestingly, 9 patients with “unknown” BC diagnosis present very similar miRNA profiles to Luminal A or B subtype BC patients (Figure 3).

**Figure 4:**
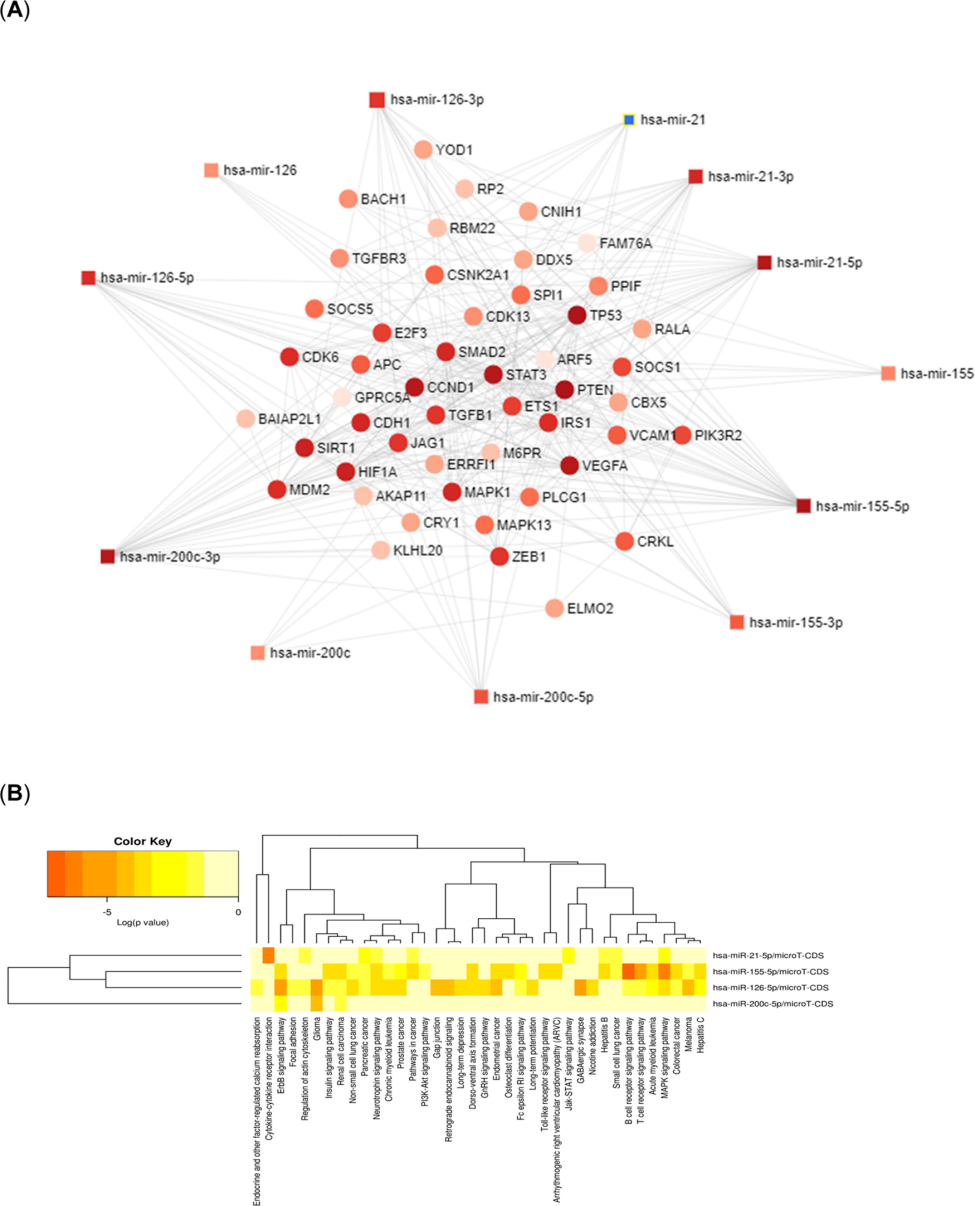
miRNAs-gene network analysis. (A) miRNA target gene network is constructed by using miRNet software (https://www.mirnet.ca). (B) The network represents signaling pathways associated with miR-21, miR-155, miR-200c, and miR-126. Representations are generated by miRPath Diana tools (DIANA TOOLS—mirPath v.3 uth.gr).

The target genes of miR-21, miR-155, miR-200c, and miR-126 were predicted by using miRNet database (http://www.mirnet.ca/). The miRNet network analysis showed that the selected miRNAs present a variable expression of downstream target genes (Figure 4A), which are associated with several biological functions. Furthermore, the signaling pathways, which are linked to the selected miRNAs (miR-21, miR-155, miR-200c, miR-126) were generated by using miRPath Diana tools (DIANA TOOLS—mirPath v.3 uth.gr) (Figure 4B). Specifically, miRPath analysis revealed that the pathways, which are associated with miR-21, miR-155, miR-200c, and miR-126 are endocrine and factor-regulated calcium reabsorption, cytokine–cytokine receptor interaction, ErbB signaling pathway, focal adhesion, regulation of actin cytoskeleton, glioma, insulin signaling pathway, renal cell carcinoma, non-small/small cell lung cancer, pancreatic cancer, neurotrophin signaling pathway, chronic/acute myeloid leukemia, prostate cancer, PI3K-Akt signaling pathway, gap junction, retrograde endocannabinoid signaling, long-term depression, dorso-ventral axis formation, GnRH signaling pathway, endometrial cancer, osteoclast differentiation, Fc epsilon RI signaling pathway, long-term potentiation, Toll-like receptor signaling pathway, arrhythmogenic right ventricular cardiomyopathy, Jak-STAT signaling pathway, GABAergic synapse, nicotine addiction, hepatitis B/C, B/T cell receptor signaling pathway, MAPK signaling pathway, colorectal cancer, and melanoma.

## Discussion

In 7 out of 48 patients (14.5%), variants were detected in the *BRCA* genes. Out of 7 patients, 5 of them carried PVs. Three of them had variants in the *BRCA2* gene, while two had variants in the *BRCA1* gene. The *BRCA1*:*BRCA2* PV ratio was determined to be 1:1.5. In three different studies performed with the Turkish BC population, the *BRCA1*:*BRCA2* PV ratios were found to be 1:2,^36^ 1:1.5,^37^ and 1.3:1,^38^ respectively. In two unrelated patients carrying a PV in the *BRCA1* gene, a heterozygous deletion in exons 18–19 was detected. This deletion represents the most common type of large genomic rearrangements in the *BRCA1* gene in individuals from Turkiye.^39^

In our study, no significant relationship was found between the *BRCA* status of patients and the age of diagnosis. Additionally, no significant relationship was found among the metastasis status, lymph node involvement, and *BRCA* status. The absence of variants in the *BRCA* genes in any of the BC patients with the Luminal A and B subtypes supports the association of these subtypes with a favorable and moderate prognosis. *BRCA1/2* pathogenic variants were detected in the cases having HER2 overexpression and TNBC phenotype. In this cohort, HER2 overexpression was present in 5 of 48 (10.4%) cases, and four of them had a pathogenic variant in one of *BRCA* genes accounting for 80% of cases having HER2 overexpression. HER2 is a member of the epidermal growth factor receptor family. It was shown that HER2 overexpression was present in 20% of BC patients and associated with poor prognosis.^40^ In our study, all (*n* = 3) *BRCA2* and 1 of 2 *BRCA1* pathogenic variant carriers had HER2 overexpression. Compared to previous data which demonstrates low frequency (ranging between 2.1 and 10%) of HER2-positive status in the BC of *BRCA1* mutation carriers, and a slightly higher rate (ranging between 6.8 and 13%) in those with mutations in *BRCA2*, our data indicates significantly high co-occurrence of HER2 overexpression and germ line *BRCA1* and 2 pathogenic variant presence.^41^ This significant difference may be due to the very small size of our cohort. It is known that approximately 15 to 25% of TNBC patients with the most aggressive behavior and worst prognosis of all BC subtypes, harbor germ line *BRCA*1/2 pathogenic variations; we found that out of six TNBC cases, one patient had a pathogenic variation in *BRCA1* gene. Although there is a significant relationship between *BRCA* and the risk of contralateral BC,^7^ in our study, no variants were detected in the *BRCA* genes in the two patients diagnosed with bilateral BC. The small sample size in our study might be a factor for this result. Detecting a PV in the *BRCA2* gene in the only patient with ipsilateral BC supports a potential relationship between *BRCA2* and ipsilateral BC. It is known that variants in *BRCA* genes increase the risk of contralateral and ipsilateral BC.^42^ However, there is no clear consensus on whether there is a difference in risk between the *BRCA1* and *BRCA2* genes. Some studies investigating the relationship between *BRCA* variants and ipsilateral BC risk have shown a potential association with *BRCA2*.^43^ Accordingly, in individuals with *BRCA2* variants, the frequency of ipsilateral BC is increased in those with multifocal BC, whereas no such relationship has been observed in individuals with *BRCA1* variants.^44^ In another study, the risk of ipsilateral BC was found to be 0.0030 in patients undergoing therapeutic nipple-sparing mastectomy with *BRCA1* variants, while in patients with *BRCA2* variants, this risk was determined to be 0.0084.^45^ More extensive research involving a larger cohort of patients is imperative to conclusively confirm and elucidate this relationship.

Moreover, metastatic BC was present in 7 of 48 cases, of which three had luminal B, two had TNBC, one had Luminal A, and one had HER2 overexpression phenotype. In only one of these TNBC cases, VUS was detected in the *BRCA2* gene. There is no clear relationship between the *BRCA2* PVs and metastasis patterns. In a previous study among 383 TNBC cases, *BRCA2* PV frequency was reported as 3.3% and it is stated that the *BRCA2* PV did not represent an independent outcome predictor of metastases. Among the Luminal ER-positive BC, Luminal A has a considerably better prognosis, and the absolute benefit from the addition of chemotherapy is minimal. The differentiation between Luminal A and B subtypes holds clinical significance in identifying a low-risk ER-positive population who could potentially avoid chemotherapy.^46^ Previous studies had shown that the rate of variant detection in the *BRCA* genes is lower in Luminal A subtype BC compared to other groups, and most variants are identified in the *BRCA2* gene.^47,48^ It was shown that patients with the Luminal A subtype have a better prognosis even if they carried *BRCA* variants, and mortality rates were quite low at the 5-year follow-up.^47^ In a recent study involving 531 BC patients, *BRCA1* variant was generally detected in the TNBC subtype, while *BRCA2* variant was specifically identified in the Luminal B subtype.^48^ In this study among 21 Luminal B BC cases, we detected two distinct variations in *BRCA2*, one of which was interpreted as pathogenic and no variant was detected in *BRCA1*. The absence of variants detected in any of the Luminal A subtype BC patients included in our study may be due to the small patient population; however, detecting fewer variants compared to other subtypes is consistent with the literature.^48,49^

In this study, we investigated the expression levels of two well-known oncomiRs, miR-21 and miR-155, which are often associated with promoting cancer progression. Concurrently, we examined the expression of miR-126 and miR-200c that typically inhibit tumor growth and metastasis. Our findings revealed distinct patterns of expression across different subtypes of BC. Notably, patients diagnosed with TNBC exhibited the highest levels of miR-21 and miR-155, both of which are commonly associated with aggressive cancer phenotypes.^8^ Patients with HER+ and Luminal A and B BC subtypes followed this. Our data suggest that miR-21 and miR-155 may play a prominent role in the molecular landscape of TNBC, potentially contributing to its aggressive nature. Conversely, the lower expression of these oncomiRs in other subtypes, such as Luminal A and B, may signify a less aggressive tumor phenotype. Moreover, we found that tumor suppressor miRNAs, miR-126 and miR-200c, expressed in the highest levels in patients with the Luminal A subtype of BC. A less aggressive phenotype often characterizes the Luminal A BC subtype, and the heightened expression of miR-126 and miR-200c in this subtype aligns with their roles as tumor suppressors, suggesting a potential protective effect. In contrast, patients with TNBC had the lowest levels of miR-126 and miR-200c expression. These data imply a potential downregulation of these protective miRNAs in TNBC, which could contribute to the aggressiveness of this subtype. Furthermore, in our study, interestingly, a subset of patients with “unknown” BC diagnosis showed similar miRNA profiles to those of either Luminal A or Luminal B subtype BC. Dysregulated expression of miR-21 and miR-155 may exacerbate the effects of *BRCA* mutations on tumorigenesis. *BRCA* mutations are involved in DNA repair mechanisms, and their dysfunction can lead to genomic instability and increased susceptibility to BC.^50^ The interplay between miR-21, miR-155, and *BRCA* mutations may further disrupt DNA repair processes, impacting genomic instability and promoting tumor development. Moreover, miR-21 and miR-155 may modulate the sensitivity of *BRCA*-mutated BC cells to therapeutic interventions, such as PARP inhibitors.^51,52^ Hence, understanding the functional implications of miR-21 and miR-155 expression patterns in the context of *BRCA* mutations is essential for developing targeted therapeutic strategies and improving patient outcomes in BC management. Further research is needed to elucidate the precise mechanisms underlying their interplay and to explore potential therapeutic interventions targeting these pathways.

To demonstrate the functional features of the selected miRNAs, miRNet was used to predict the target genes of miR-21, miR-155, miR-200c, and miR-126. In miRNet analysis, results showed that *BRCA* genes are not targets of miR-21, miR-155, miR-200c, and miR-126 in BC. This is in line with our study’s results, which revealed no correlation between the expression levels of the selected miRNAs and *BRCA* status. Moreover, miRNet analysis revealed that some of the signaling pathways related to the examined miRNAs in this study are PTEN, TP53, HIF1A1, TGF-β (*SMAD2*, *TGFB1*, *TGFBR3*), STAT (*STAT3*), MAPK (*MAPK13*, *MAPK1*), VEGF (*VEGFA*), and PI3K (*PIK3R2*). It has been previously reported that *PTEN* is an important target gene of miR-21, which can inhibit apoptosis and promote tumor cell growth, metastasis, and invasion.^53^ Other studies on prostate cancer demonstrated that miR-21 overexpression induces PI3K/Akt signaling pathway, which is involved in cell growth, survival, and metabolism and increases HIF-1α and VEGF expression, then induces tumor angiogenesis.^54^ Recent study has shown that high expression of miR-155 promotes BC progression and involves in paclitaxel resistance via *TP53INP1*.^55^ Upregulation of miRNA-155 promotes tumor angiogenesis by targeting VHL and is associated with poor prognosis and TNBC.^56^ miR-155 was also shown to target PTEN, leading to its downregulation, and this contributes to increased PI3K/Akt signaling and oncogenic processes.^57^ Expression level of miR-155 was found to be closely related to the status of the ER and PGR.^58^ Furthermore, miR-155 has been reported to target STATs in certain contexts, affecting downstream signaling pathways involved in cell proliferation, survival, and immune response. Dysregulation of miR-155-mediated STAT regulation may contribute to cancer progression and resistance to therapy.^59^ It has been demonstrated that miR-126 can moderate angiogenesis through inhibiting *VEGFA* in BC.^60^ miRNet analysis also indicated that *ZEB1* is a target gene of miR-200c in BC. A previous study has also illustrated that miR-200c can inhibit stemness and promote the cellular sensitivity to trastuzumab in HER2+ BC cells via *ZEB1*.^61^ Moreover, it was reported that miR-200c increases radiosensitivity of various human cancer cells including TNBC cell line MDA-MB-231, via activated EGFR-associated signaling.^62^ miR-200c downregulation results in enhanced metastasis in BC and it is a known target of TP53 gene, which regulates stemness.^63^ In this study, miRPath analysis showed that several signaling pathways such as the ErbB signaling pathway are linked to miR-21, miR-155, miR-126, and miR-200c. It has been suggested previously that ErbB receptors are highly expressed or mutated in several malignancies, especially in BC, ovarian cancer, and non-small-cell lung cancer. The overexpression of ErbB receptors is related with poor prognosis, drug resistance, metastasis, and lower survival rate in BC.^64^ Moreover, in this study, miRPath analysis indicated that the PI3K-Akt signaling pathway and MAPK signaling pathways are correlated to miR-21, miR-155, miR-126, and miR-200c. Interactions between the PI3K/AKT/mTOR pathway and the BRCA pathway have been reported, suggesting potential crosstalk that influences tumor behavior. Activating mutations in the PIK3CA gene leads to hyperactivation of PI3K pathway.^65^ It was reported that mutations in *PI3KCA* are more common in luminal A subtype cancers (45% of cases), followed by HER2+ mutations (39%), luminal B (30%), and triple-negative BC alterations in 9% of cases.^66^ These mutations are crucial in BC, as approximately 27% of patients exhibiting mutations in this gene.^67^ Therefore, PI3K activation plays a crucial role in BC development and therapeutic resistance in ER+/HER2+ BC cases.^68^ Furthermore, the MAPK signaling pathway is an important signal transduction pathway associated with invasion metastasis and prognosis in TNBC cases.^69−71^ Deregulated TGF-β signaling is associated with BC progression, and its interaction with the BRCA pathway may impact tumor aggressiveness and therapy response.^72^ TP53 mutations may intersect with the BRCA pathway, influencing tumor development and therapeutic outcomes.^73^ Understanding the relevance of these pathways to BC progression and their potential intersection with the *BRCA* pathway is crucial for identifying novel therapeutic targets and improving patient outcomes. Integration of miRNet analysis provides valuable insights into the complex network of genes and pathways involved in BC pathogenesis, facilitating the development of more targeted and effective treatment strategies.

Although this study adds significant and useful information to the current knowledge in the field, it does, however, show some potential limitations. The most important limiting factor of our study is the relatively low number of patients, considering the prevalence of BC. Although our results are consistent with the literature, studies with larger sample sizes and meta-analyses are needed for generalization. The relationship between *BRCA2* and ipsilateral breast cancer, which is one of the intriguing findings in our study, should be further investigated in future research. Additionally, our study, which investigates the relationship between miRNA and BC subtypes/*BRCA* genes, needs to be supported by other studies. Thus, the utility of miRNAs as reliable biomarkers can be determined.

## Conclusions

In conclusion, our findings present strong evidence, suggesting that circulating miRNAs, specifically miR-21, miR-155, miR-126, and miR-200c, have the potential to serve as diagnostic markers for BC and its subtypes, corresponding to their metastatic capabilities. Notably, our analysis indicates that *BRCA* status does not correlate strongly with BC’s metastatic status. However, a significant relationship between *BRCA1/2* PV presence and poor prognostic histopathological subtypes was determined. Moreover, in our study, *BRCA* mutations were not correlated to miRNA expression patterns. The role of miRNAs in the onset and advancement of BC holds significant promise for innovative advancements in diagnostic and therapeutic approaches for BC management. Existing evidence suggests a connection between *BRCA* mutations and altered miRNA expressions in BC, highlighting miRNAs’ potential role in hereditary BC susceptibility. The relationship between *BRCA* mutations and miRNA expression in BC is complex and likely involves multiple interacting factors. Further investigations are needed to understand these interactions’ precise molecular mechanisms and clinical implications.

## Methodology

### Ethics

An informed consent form was obtained from all of the patients included in this study. In addition, appropriate genetic counseling was given to all patients before and after genetic testing. Ethical permission for the conduction of the study was obtained from the institutional ethics committee (Marmara University, Medical School, Ethics Committee 434/030323).

### Genetic Testing and DNA Extraction

Detailed clinical information and medical reports were collected from all patients, which is summarized in Table 2. Patients diagnosed with BC were included in the study. DNA isolation from peripheral blood was performed with the QIAamp DNA Mini Kit (Qiagen, MD). *BRCA1* and *BRCA2* genes were amplified via Multiplicom BRCAMaster Dx (Agilent, CA). To detect gross deletion/duplications, the SALSA multiplex ligation-dependent probe amplification (MLPA) Probemix P002 BRCA1 and P045 BRCA/CHEK2 kits (MRC Holland, Amsterdam, The Netherlands) were used. Sequencing was performed using the Illumina NextSeq platform (Illumina, Inc., San Diego, CA). The data were analyzed in the SophiaDDM (Sophia Genetic, Inc. Boston, MA 02116). Pathogenicity of the variants was evaluated according to the American College of Medical Genetics and Genomics (ACMG) criteria.^74^

**Table 2:** Patient Clinical Characteristics

characteristics	number of patients, *n* (%)
age range: 33–73	
median age: 44.45	
ER status	
positive	33 (68.7%)
negative	11 (22.9%)
unknown	4 (8.3%)
PR Status	
positive	29 (60.4%)
negative	15 (31.2%)
unknown	4 (8.3%)
HER-2 Status	
positive	13 (27%)
negative	31 (64.5%)
unknown	4 (8.3%)
Tumor Size	
Tis	0
T1	19 (39.5%)
T2	14 (29%)
T3	0
T4	1 (2%)
unknown	14 (29%)
Lymph Nodes	
N0	16 (33.3%)
N1	9 (18.7%)
N2	7 (14.5%)
N3	3 (6.2%)
unknown	13 (27%)
Metastasis	
M0	28 (58.3%)
M1	7 (14.5%)
unknown	13 (27%)
Histological Tumor Grade	
Tis (0)	0
I	3 (6.2%)
II	8 (16.6%)
III	4 (8.3%)
IV	0
unknown	33 (68.7%)
Molecular Subtypes	
HER-2 overexpression	5 (10.4%)
luminal A	7 (14.5%)
luminal B	21 (43.7%)
triple negative	6 (12.5%)
unknown	9 (18.7%)

### RNA Extraction and RT-qPCR

RNA was extracted from BC blood samples using Trizol (Sigma, Haverhill, U.K.), and RNA concentration and purity were measured using the NanoDrop spectrophotometer (Thermo Fisher Scientific, Hemel Hempstead, U.K.) at 260 and 280 nm absorbance. Reverse transcription of RNA to cDNA was carried out using a miRCURY LNA RT Kit (Qiagen, Manchester, U.K.) according to the manufacturer’s instructions. The miRCURY LNA miRNA SYBR Green (Qiagen, Manchester, U.K.) was used in conjunction with MystiCq microRNA qPCR primers for miR-21, miR-155, miR-126, and miR-200c (Sigma, Haverhill, U.K.). The expression levels of miRNAs were normalized to that of U6 using the 2^∧^ΔΔCT method.^75^ The sequences for U6 primers were forward 5′-GCTTCGGCAGCACATATACTAAAAT-3′ and reverse 5′- CGCTTCACGAATTTGCGTGTCAT-3′. The RT-qPCR conditions were as follows: heat activation at 95 °C for 2 min, followed by 40 cycles at denaturation at 95 °C for 10 s and combined annealing/extension at 56 °C for 60 s.

### miRNA–Target Interaction Networks

miRNet was used for the identification of novel gene connections among the selected miRNAs and visualization of miR-target gene interaction networks. Interaction networks were built based on organism choice “*Homo Sapiens*”, ID type “miRBase ID”, Tissue “Breast Cancerous Tissue”, Targets “miRTarBase, TarBase, miRecords” in miRNet software. The miRNet version 2 Web site is freely available at https://www.mirnet.ca.^76,77^ miRNA-related signaling pathways were analyzed by using DIANA tools mirPath (DIANA TOOLS—mirPath v.3 -uth.gr).^78^

### Data Analysis

All data were analyzed as mean ± standard deviation. Results were considered significant for *p* < 0.05. One-way ANOVA Bonferroni’s multiple comparisons test was performed using GraphPad Prism version 9.3.1 for Windows (GraphPad Software, La Jolla, CA) www.graphpad.com.
